# Risk of thyroid dysfunction associated with mRNA and inactivated COVID-19 vaccines: a population-based study of 2.3 million vaccine recipients

**DOI:** 10.1186/s12916-022-02548-1

**Published:** 2022-10-14

**Authors:** Carlos King Ho Wong, David Tak Wai Lui, Xi Xiong, Celine Sze Ling Chui, Francisco Tsz Tsun Lai, Xue Li, Eric Yuk Fai Wan, Ching Lung Cheung, Chi Ho Lee, Yu Cho Woo, Ivan Chi Ho Au, Matthew Shing Hin Chung, Franco Wing Tak Cheng, Kathryn Choon Beng Tan, Ian Chi Kei Wong

**Affiliations:** 1 Centre for Safe Medication Practice and Research, Department of Pharmacology and Pharmacy, Li Ka Shing Faculty of Medicine, The University of Hong Kong, Hong Kong SAR, China; 2 Department of Family Medicine and Primary Care, School of Clinical Medicine, Li Ka Shing Faculty of Medicine, The University of Hong Kong, Hong Kong SAR, China; 3 Laboratory of Data Discovery for Health (D24H), Hong Kong Science and Technology Park, Hong Kong SAR, China; 4 Department of Medicine, School of Clinical Medicine, Li Ka Shing Faculty of Medicine, The University of Hong Kong, Hong Kong SAR, China; 5 School of Nursing, Li Ka Shing Faculty of Medicine, The University of Hong Kong, Hong Kong SAR, China; 6 School of Public Health, Li Ka Shing Faculty of Medicine, The University of Hong Kong, Hong Kong SAR, China; 7 Research Department of Practice and Policy, UCL School of Pharmacy, University College London, London, UK; 8 Aston School of Pharmacy, Aston University, Birmingham, UK

**Keywords:** COVID-19 vaccines, BNT162b2 vaccine, Vaccines, Inactivated, Hyperthyroidism, Hypothyroidism, Graves’ disease, Thyroiditis

## Abstract

**Background:**

In view of accumulating case reports of thyroid dysfunction following COVID-19 vaccination, we evaluated the risks of incident thyroid dysfunction following inactivated (CoronaVac) and mRNA (BNT162b2) COVID-19 vaccines using a population-based dataset.

**Methods:**

We identified people who received COVID-19 vaccination between 23 February and 30 September 2021 from a population-based electronic health database in Hong Kong, linked to vaccination records. Thyroid dysfunction encompassed anti-thyroid drug (ATD)/levothyroxine (LT4) initiation, biochemical picture of hyperthyroidism/hypothyroidism, incident Graves’ disease (GD), and thyroiditis. A self-controlled case series design was used to estimate the incidence rate ratio (IRR) of thyroid dysfunction in a 56-day post-vaccination period compared to the baseline period (non-exposure period) using conditional Poisson regression.

**Results:**

A total of 2,288,239 people received at least one dose of COVID-19 vaccination (57.8% BNT162b2 recipients and 42.2% CoronaVac recipients). 94.3% of BNT162b2 recipients and 92.2% of CoronaVac recipients received the second dose. Following the first dose of COVID-19 vaccination, there was no increase in the risks of ATD initiation (BNT162b2: IRR 0.864, 95% CI 0.670–1.114; CoronaVac: IRR 0.707, 95% CI 0.549–0.912), LT4 initiation (BNT162b2: IRR 0.911, 95% CI 0.716–1.159; CoronaVac: IRR 0.778, 95% CI 0.618–0.981), biochemical picture of hyperthyroidism (BNT162b2: IRR 0.872, 95% CI 0.744–1.023; CoronaVac: IRR 0.830, 95% CI 0.713–0.967) or hypothyroidism (BNT162b2: IRR 1.002, 95% CI 0.838–1.199; CoronaVac: IRR 0.963, 95% CI 0.807–1.149), GD, and thyroiditis. Similarly, following the second dose of COVID-19 vaccination, there was no increase in the risks of ATD initiation (BNT162b2: IRR 0.972, 95% CI 0.770–1.227; CoronaVac: IRR 0.879, 95%CI 0.693–1.116), LT4 initiation (BNT162b2: IRR 1.019, 95% CI 0.833–1.246; CoronaVac: IRR 0.768, 95% CI 0.613–0.962), hyperthyroidism (BNT162b2: IRR 1.039, 95% CI 0.899–1.201; CoronaVac: IRR 0.911, 95% CI 0.786–1.055), hypothyroidism (BNT162b2: IRR 0.935, 95% CI 0.794–1.102; CoronaVac: IRR 0.945, 95% CI 0.799–1.119), GD, and thyroiditis. Age- and sex-specific subgroup and sensitivity analyses showed consistent neutral associations between thyroid dysfunction and both types of COVID-19 vaccines.

**Conclusions:**

Our population-based study showed no evidence of vaccine-related increase in incident hyperthyroidism or hypothyroidism with both BNT162b2 and CoronaVac.

**Supplementary Information:**

The online version contains supplementary material available at 10.1186/s12916-022-02548-1.

## Background

The coronavirus disease 2019 (COVID-19) pandemic, caused by the severe acute respiratory syndrome coronavirus 2 (SARS-CoV-2), has infected close to 500 million people and caused about 6.1 million deaths [1]. COVID-19 vaccination is essential in bringing the COVID-19 pandemic under control and minimising the serious health consequences among COVID-19 patients [2]. Studies have evaluated the inter-relationship between COVID-19 and thyroid dysfunction [3]: non-thyroidal illness syndrome, thyroiditis, Graves’ disease (GD), and Hashimoto’s thyroiditis. The onset of GD and Hashimoto’s thyroiditis after the diagnosis of COVID-19 raised the concern of SARS-CoV-2 in inducing autoimmune thyroid disorders [4, 5]. This postulation is supported by the expression of angiotensin-converting enzyme 2 (ACE2), the functional receptor for SARS-CoV-2, in many endocrine organs, including the thyroid [6].

It remains to be determined whether COVID-19 vaccination may be associated with incident thyroid dysfunction by the same token. CoronaVac is an example of inactivated vaccines, which contain an inactivated form of SARS-CoV-2. On the other hand, BNT162b2 (equivalent to Pfizer-BioNTech mRNA vaccine) stimulates the production of SARS-CoV-2 spike protein. These may in turn interact with the thyroid via ACE2. Indeed, there is an increasing number of case reports of incident thyroid dysfunction after COVID-19 vaccination [7]. These include cases of subacute thyroiditis after various COVID-19 vaccines, GD mostly after mRNA [7] and occasionally after adenovirus-vectored vaccine [8], and recently overt hypothyroidism after mRNA vaccine [9]. Molecular mimicry and autoimmune/inflammatory syndrome induced by adjuvants (ASIA) are the postulated mechanisms for thyroid dysfunction with clinical manifestations of hyperthyroidism or hypothyroidism after COVID-19 vaccination [9–11]. A recent study has demonstrated the cross-reactivity of SARS-CoV-2 spike protein, nucleoprotein, and membrane protein with thyroid peroxidase, and the sequence homology/similarity between many thyroid peroxidase peptide sequences and sequences in various SARS-CoV-2 proteins [10]. Furthermore, the adjuvants in the vaccines, such as the aluminium salts in CoronaVac, may have the potential to cause ASIA [11].

Current knowledge about the possible link between COVID-19 vaccination and thyroid dysfunction is limited to case reports and series. Also, thyroid dysfunction has not been comprehensively captured in the vaccine surveillance databases. While more than 11 billion doses of COVID-19 vaccines have been administered as of 1 April 2022, there are more than 80 cases of COVID-19 vaccination-associated thyroid dysfunction reported in the literature [12]. However, case reports and series do not quantify the absolute risk of thyroid dysfunction and inform if COVID-19 vaccination is associated with an increased risk of thyroid dysfunction. Moreover, clinical trials of COVID-19 vaccines have not reported thyroid-specific outcomes in detail. Such information is essential to inform clinical practice for advising patients for COVID-19 vaccination and the subsequent follow-up or surveillance. The Hong Kong Government Vaccination Programme currently provides two authorised COVID-19 vaccines: CoronaVac (inactivated whole-virus vaccine) and BNT162b2 (mRNA vaccine). We conducted this population-based study to evaluate the risks of incident thyroid dysfunction associated with these two types of COVID-19 vaccination, using the self-controlled case series (SCCS) design.

## Methods

### Data source

Since 23 February 2021, the COVID-19 Vaccination Programme has been launched in Hong Kong, and vaccination records have been reported to the Department of Health (DH) daily. All active patients’ electronic medical records (EMRs) between 1 January 2018 and 30 September 2021 were extracted from the Hong Kong Hospital Authority (HA) and cross-linked with vaccination records by hashed unique identifiers. This vaccine safety data linkage is largely representative of the Hong Kong population as HA is the only provider of public healthcare services, which provides medical services for 90% of all primary, secondary, and tertiary care services of Hong Kong with a population of more than 7 million [13]. EMRs provided by HA are generated during public healthcare services. Available information includes demographics, date of registered death, drug dispensing records, diagnoses, procedures, and laboratory tests. All prescriptions dispensed by HA pharmacies are recorded. These two linked data sources have been recently used to conduct population-based pharmacovigilance studies of COVID-19 vaccines [14–27].

The study received ethical approval from the Institutional Review Board of the University of Hong Kong/Hospital Authority Hong Kong West Cluster (UW 21-149 and UW 21-138); and the Department of Health Ethics Committee (LM 21/2021).

### Study design

We conducted an SCCS study to evaluate the associations between COVID-19 vaccines and incident thyroid dysfunction during the COVID-19 Vaccination Programme in Hong Kong. SCCS was designed to evaluate the relative incidence of outcome events at defined risk periods compared with baseline periods among people with outcome events during the observation period [28]. The advantages of this study design are that individuals’ comparison removes the potential confounding effect of all time-invariant variables during the study period. This methodology has been used to estimate COVID-19 vaccine safety in neurological, thrombocytopenic, thromboembolic and haemorrhagic events, haematological disorders, and herpes zoster-related hospitalizations [19, 20, 29–32].

### Study population

People aged ≥ 18 years who used any HA services between 1 January 2018 and 30 September 2021 were identified from the vaccine safety data linkage. We excluded people who (i) received a heterologous COVID-19 vaccine regime and (ii) were polymerase chain reaction-tested positive for SARS-CoV-2 from 23 January 2020 (the first COVID-19 case in Hong Kong) to 30 September 2021. We also excluded people who had history of ATD and LT4 prescription records, abnormal thyroid-stimulating hormone (TSH) levels, GD, or thyroiditis within the 3 years before the observation period.

### Outcomes of interest: thyroid dysfunction

Study outcomes were initiation of ATD, initiation of LT4, hyperthyroidism, hypothyroidism, GD, and thyroiditis. We identified ATD and LT4 prescriptions using British National Formulary (BNF) code and drug names from EMRs (ATD: BNF 6.2.2; LT4: BNF 6.2.1). Initiation of ATD was taken to represent incident hyperthyroidism, while initiation of LT4 was taken to represent incident hypothyroidism. Hyperthyroidism was defined by TSH < 0.35 mIU/L, while hypothyroidism by TSH > 4.8 mIU/L. The International Classification of Diseases-9 (ICD-9) codes was used to identify GD (ICD-9: 242.00 and 242.01) and thyroiditis (ICD-9: 245.x).

### Exposure

**Fig. 1:**
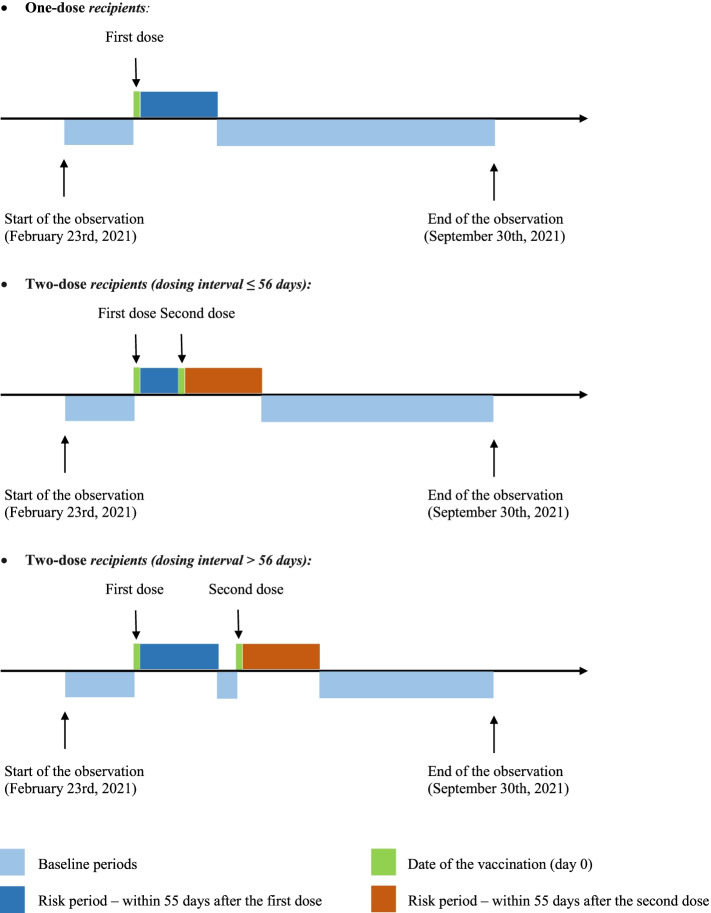
Schematic presentation of the observation period of COVID-19 vaccine recipients in the self-controlled case series study

The observation period was from 23 February 2021 to 30 September 2021. The exposure was the first or second dose of CoronaVac or BNT162b2 vaccines. Based on reviews of published case reports on subacute thyroiditis and GD after COVID-19 vaccination showing that the onset of thyroid dysfunction could occur from several days to up to 8 weeks post-vaccination [33, 34], the exposure period was defined as a risk window of 56 days post-vaccination. For people who received the second dose before day 56, the period before the second dose was considered as post-first dose exposure, while the period after the second dose was considered as post-second dose exposure. The remaining periods during the observation period were classified as the baseline period (non-exposure period). A typical observation period of a vaccinated individual is shown in Fig. 1.

### Statistical analyses

#### Incidence of thyroid dysfunction

For CoronaVac and BNT162b2 recipients, the number of events per 100,000 doses, and crude incidence rates per 100,000 person-years after the first and second doses was estimated. The follow-up period began from the date of each dose and was censored on the date of death, 56 days, end of the study period, or date of the second dose (for first dose recipients), whichever occurred first.

#### SCCS analysis

The SCCS model was applied to estimate the incidence rate ratios (IRRs) with the corresponding 95% confidence intervals (CI) of each outcome. Whether the SCCS models are appropriate relies on three key assumptions. (i) Recurrent events must be independent. The occurrence of the first event should not influence the likelihood of the subsequent event. Thus, we only treat the first episode as the outcome of interest in our analysis [35]. (ii) Occurrence of an event should not alter the probability of subsequent exposure. Histograms of the number of events by the interval between the date of each dose (exposure date) and event day are illustrated in Additional file 1: Fig. S1. For example, if patients were initiated on ATD before vaccination, they might be less likely to receive the vaccines. It would lead to a low rate of events right before vaccination and increase the relative rate of events occurring in the risk period versus the baseline period. (iii) Event should not censor observation periods. Thus, we conducted the analysis using the modified SCCS model “eventdepenexp” in the R-package “SCCS” [36]. The use of the modified SCCS in COVID-19 vaccine research has been comprehensively discussed in a recent publication [37]. The modified SCCS requires including unvaccinated people during the observation period to inform the timing of the events by adjusting for the monthly seasonal effects.

Subgroup analyses were conducted by age (< 65 vs ≥ 65 years) among men and women separately due to different sex distributions between the younger and older. Sensitivity analyses were conducted (i) by varying the risk periods from the first dose to 42, 35, 28, and 21 days after the first dose, or from the second dose to 42, 35, 28, and 21 days after the second dose, and (ii) by excluding the pre-vaccination period due to the possible increase in diagnosis of thyroid dysfunction when the vaccinated individuals had thyroid function tests prior to vaccination.

Two-sided *p* values of < 0.05 were considered statistically significant. Statistical analyses were independently conducted and cross-checked by co-authors (XX, MSHC, ICHA, and FWTC) for quality assurance. All analyses were performed in R version 4.1.0 (R Foundation for Statistical Computing, Vienna, Austria) and Stata Version 16.0 (StataCorp LP, College Station, TX).

## Results

A total of 1,321,753 and 966,486 people received at least one dose of BNT162b2 and CoronaVac, respectively, from 23 February to 30 September 2021 (Additional file 1: Fig. S2). 94.3% of BNT162b2 recipients and 92.2% of CoronaVac recipients completed two doses of vaccine within the study period. The mean age, Charlson comorbidity index, and proportion of men were 45.6 years (SD: 16.1), 1.4 (SD: 1.5) and 45.1% in the BNT162b2 group, and 55.4 (SD: 14.2), 2.2 (SD: 1.5) and 46.5% in the CoronaVac group, respectively. Baseline characteristics of vaccinated people who developed outcomes of ATD initiation, LT4 initiation, incident hyperthyroidism, incident hypothyroidism, GD, and thyroiditis are summarised in Additional file 1: Table S1.

### Incidence of thyroid dysfunction

Regarding thyrotoxicosis-related outcomes, the incidences of biochemical hyperthyroidism, ATD initiation, GD, and thyroiditis were 35.4, 22.6, 1.6, and 0.5 cases per 100,000 doses within 56 days following the first dose of BNT162b2; 65.9, 41.4, 1.8, and 0.4 cases per 100,000 doses following the second dose of BNT162b2; 47.4, 25.1, 0.4, and 0.4 cases per 100,000 doses following the first dose of CoronaVac; and 73.5, 46.0, 1.9, and 0.7 cases per 100,000 doses following the second dose of CoronaVac (Additional file 1: Table S2).

Regarding hypothyroid-related outcomes, the incidences of biochemical hypothyroidism and LT4 initiation were 26.5 and 21.3 cases per 100,000 doses following the first dose of BNT162b2, 46.9 and 48.2 cases per 100,000 doses following the second dose of BNT162b2, 36.8 and 33.2 cases per 100,000 doses following the first dose of CoronaVac, and 52.0 and 49.5 cases per 100,000 doses following the second dose of CoronaVac.

### Risk of thyroid dysfunction

Table 1 summarises the outcomes of thyroid dysfunction among the vaccine recipients. The IRRs for thyrotoxicosis-related outcomes of interest compared to the baseline period indicated no significant increase in the risk of biochemical hyperthyroidism (BNT162b2: IRR = 0.872, 95% CI: 0.744-1.023; CoronaVac: IRR = 0.830, 95% CI: 0.713–0.967) and initiation of ATD (BNT162b2: IRR = 0.864, 95% CI: 0.670–1.114; CoronaVac: IRR = 0.707, 95% CI: 0.549–0.912) related to the first doses of COVID-19 vaccine. There was no significant increase in the incidence of GD or thyroiditis following the first dose of either type of COVID-19 vaccination. The IRRs for these outcomes of interest compared to the baseline period indicated no significant increase in the risk of biochemical hyperthyroidism (BNT162b2: IRR = 1.039, 95% CI: 0.899–1.201; CoronaVac: IRR = 0.911, 95% CI: 0.786–1.055), initiation of ATD (BNT162b2: IRR = 0.972, 95% CI: 0.770–1.227; CoronaVac: IRR = 0.879, 95% CI: 0.693–1.116) related to the second doses of COVID-19 vaccine. There was no significant increase in the incidence of GD or thyroiditis following the second dose of either type of COVID-19 vaccination.

**Table 1:** Risks of thyroid dysfunction in the 56-day risk period following the first or second dose of COVID-19 vaccination

Outcomes	CoronaVac				BNT162b2			
	No. of event	Person years	IRR	95% CI	No. of event	Person years	IRR	95% CI
**Initiation of ATD**								
Baseline	3116	1878.3	1.00	-	3299	2000.0	1.00	-
0 to 55 days: first dose	109	74.5	0.707	(0.549, 0.912)	130	76.4	0.864	(0.670, 1.114)
0 to 55 days: second dose	182	99.3	0.879	(0.693, 1.116)	216	119.1	0.972	(0.770, 1.227)
**Initiation of LT4**								
Baseline	5315	3131.6	1.00	-	5575	3280.4	1.00	-
0 to 55 days: first dose	167	125.6	0.778	(0.618, 0.981)	144	118.6	0.911	(0.716, 1.159)
0 to 55 days: second dose	217	175.4	0.768	(0.613, 0.962)	288	219.2	1.019	(0.833, 1.246)
**Hyperthyroidism (TSH < 0.35 mIU/L)**								
Baseline	11423	6896.8	1.00	-	11781	7141.6	1.00	-
0 to 55 days: first dose	354	214.5	0.830	(0.713, 0.967)	345	201.5	0.872	(0.744, 1.023)
0 to 55 days: second dose	505	286.5	0.911	(0.786, 1.055)	619	333.6	1.039	(0.899, 1.201)
**Hypothyroidism (TSH > 4.8 mIU/L)**								
Baseline	8707	5250.1	1.00	-	8941	5419.6	1.00	-
0 to 55 days: first dose	300	172.3	0.963	(0.807, 1.149)	307	158.9	1.002	(0.838, 1.199)
0 to 55 days: second dose	380	231.6	0.945	(0.799, 1.119)	463	270.7	0.935	(0.794, 1.102)
**Graves’ disease**								
Baseline	288	171.9	1.00	-	325	199.5	1.00	-
0 to 55 days: first dose	3	5.2	NA	NA	21	9.0	1.105	(0.534, 2.289)
0 to 55 days: second dose	15	7.2	NA	NA	21	12.6	0.823	(0.424, 1.599)
**Thyroiditis**								
Baseline	60	37.0	1.00	-	60	38.2	1.00	-
0 to 55 days: first dose	4	2.1	NA	NA	6	1.7	NA	NA
0 to 55 days: second dose	6	3.0	NA	NA	5	2.9	NA	NA

*ATD* anti-thyroid drug, *LT4* levothyroxine, *IRR* incidence rate ratio, *CI* confidence interval, *TSH* thyroid-stimulating hormone, *NA* not available if the number of events in one of the risk periods were ≤ 5

The IRRs for hypothyroid-related outcomes of interest compared to the baseline period indicated no significant increase in the risk of biochemical hypothyroidism (BNT162b2: IRR = 1.002, 95% CI: 0.838–1.199; CoronaVac: IRR = 0.963, 95% CI: 0.807–1.149), initiation of LT4 (BNT162b2: IRR = 0.911, 95% CI: 0.716–1.159; CoronaVac: IRR = 0.778, 95% CI: 0.618–0.981) related to the first doses of COVID-19 vaccine. The IRRs for these outcomes of interest compared to the baseline period indicated no significant increase in the risk of hypothyroidism-related outcomes related to both doses of COVID-19 vaccines. The IRRs for these outcomes of interest compared to the baseline period indicated no significant increase in the risk of biochemical hypothyroidism (BNT162b2: IRR = 0.935, 95% CI: 0.794–1.102; CoronaVac: IRR = 0.945, 95% CI: 0.799–1.119) and initiation of LT4 (BNT162b2: IRR = 1.019, 95% CI: 0.833–1.246; CoronaVac: IRR = 0.768, 95% CI: 0.613–0.962) related to the second doses of COVID-19 vaccine.

The results of the subgroup and sensitivity analyses were consistent with the main analysis (Additional file 1: Tables S3-S9). There was no change in the results when the risk period was shortened to as early as 3 weeks post-vaccination.

## Discussion

This is the first population-based study of the risk of incident thyroid dysfunction associated with COVID-19 vaccination. We did not observe any major signal of increased risks of incident thyroid dysfunction within 56 days of the first and second doses of both mRNA and inactivated COVID-19 vaccines. Thyroid dysfunction following COVID-19 vaccination was rare.

Following the report of the first cases of GD post-COVID-19 vaccination in May 2021 [38], many case reports of various thyroid dysfunction have been published: not limited to GD, but also subacute thyroiditis and hypothyroidism. The temporal relationship was the main reason to believe that these events were related to COVID-19 vaccination. Invariably, the commonly postulated mechanisms linking COVID-19 vaccination and thyroid dysfunction for all these cases included molecular mimicry [10], and ASIA [11]. Nonetheless, case reports or series are not able to estimate absolute risks and examine association between COVID-19 vaccination and thyroid dysfunction. Our population-based SCCS analysis suggested no excess in risk of GD in the 56 days following the first and second doses of either type of COVID-19 vaccination, reflected in several ways in our results. Firstly, we did not observe an increased risk of biochemical diagnosis of thyrotoxicosis. Thyrotoxicosis is most commonly due to primary hyperthyroidism and, less commonly, destructive thyroiditis. GD accounts for 60–80% of cases of primary hyperthyroidism [39]. As GD incidence varies with age and sex (peaks at 30–50 years of age and is more common in females) [40], we performed age- and sex-specific subgroup analyses, which consistently showed the neutral association between COVID-19 vaccination and the risks of thyrotoxicosis. Secondly, there was no increase in the rate of ATD initiation after COVID-19 vaccination was observed. ATD initiation largely represents the occurrence of incident GD, since GD is the most common aetiology of primary hyperthyroidism. Thirdly, the incidence of diagnostic coding of GD was not increased in association with COVID-19 vaccination. In line with our findings, a recent case series reported that most patients who developed GD after the first dose of COVID-19 vaccination received the subsequent dose of vaccination without instability in thyroid function [41]. Furthermore, all these cases of COVID-19 vaccine-related GD share similar features as the typical GD seen in our clinical practice—in terms of demographics, clinical features, and management. All cases of GD post-vaccination were easily controlled with ATD [34]. All these suggested that the reported cases of GD following COVID-19 vaccination may merely be coincidence in timing, while the preponderance of GD associated with mRNA vaccination may be due to the higher coverage of that type of vaccine [12].

In addition, our study did not suggest an increased risk of thyroiditis associated with COVID-19 vaccination, as we did not observe an increased risk of biochemical diagnosis of hyperthyroidism, nor an increased risk of diagnostic coding of thyroiditis. A review of the available cases showed no predilection of the occurrence of subacute thyroiditis after the first and second dose of COVID-19 vaccines [42]. A recent case series showed that patients who developed subacute thyroiditis after COVID-19 vaccination proceeded to the subsequent dose without recurrence [43]. Furthermore, a single-centre analysis in Turkey revealed no significant increase in the number of events of subacute thyroiditis before and after commencement of the nationwide COVID-19 vaccination programme [42]. Last but not least, a recent study of 72 healthy individuals in Greece showed no clinically significant change in thyroid function, anti-thyroid peroxidase and anti-thyroglobulin antibody titres up to one month after the second dose of mRNA COVID-19 vaccines [44]. Hence, there is yet strong evidence to support the causal relationship between subacute thyroiditis and COVID-19 vaccination.

Our study also showed no increase in the incidence of biochemical diagnosis of hypothyroidism (comprising both subclinical and overt hypothyroidism) and the LT4 initiation (reflecting clinically significant hypothyroidism). Indeed, among the many reports of thyroid dysfunction following COVID-19 vaccination, there was only one report of overt hypothyroidism following mRNA vaccination [9]. Together with the abovementioned cohort study performed in Greece [44], all these reassure that COVID-19 vaccination does not lead to hypothyroidism.

Our study has several strengths. Firstly, it is the first study to quantify the risk of thyroid dysfunction following COVID-19 vaccination using a population-based dataset. Secondly, we analysed the risk of thyroid dysfunction among recipients of two different types of COVID-19 vaccines. Thirdly, the modified SCCS model minimised the measured or unmeasured time-invariant confounders and ensured that the analysis did not violate the assumption of independence between outcome and exposure. Our results showed that the risk of thyroid dysfunction following COVID-19 vaccination was not higher than in the baseline period. Vaccinated individuals in Hong Kong might have thyroid function tests and health checks prior to the vaccination and have a higher chance of detecting hyperthyroidism and hypothyroidism or initiation ATD and LT4 during the pre-vaccination period. We demonstrated the robustness of our results by removing the pre-vaccination period in the sensitivity analysis, showing consistent results (Additional file 1: Table S9). However, our results should be interpreted bearing certain limitations. Firstly, given the limitations of the electronic health database, GD and thyroiditis were identified using diagnostic codes and inferred by the biochemical diagnosis of hyperthyroidism and initiation of ATD. Anti-thyroid antibodies were not systematically evaluated. Ultrasonographic and scintigraphic features were not available in our dataset. Hence, the incidence of GD might be underestimated. However, it was unlikely to affect our conclusion because of the nature of SCCS design comparing incidence rate of thyroid dysfunction between post-vaccination and baseline periods. Secondly, using TSH to capture thyroid dysfunction might have included cases of secondary hyperthyroidism and hypothyroidism, though these were expected to be uncommon. Thirdly, people who did not seek medical attention for subclinical thyroid problems might not be captured. Nevertheless, only clinically overt thyroid dysfunction is relevant and concerns potential COVID-19 vaccine recipients. Last but not least, the risk of GD associated with the heterologous COVID-19 vaccination regime is not addressed in our current study. Three cases of GD were reported after a booster dose (two mRNA vaccines and one adenovirus-vectored vaccine) following two doses of inactivated COVID-19 vaccines [43, 45]. Future surveillance data are warranted to specifically evaluate the potential of heterologous COVID-19 vaccination in inducing GD.

## Conclusions

Despite a large number of case reports on thyroid dysfunction following COVID-19 vaccination, our study showed that CoronaVac (inactivated whole-virus vaccine) and BNT162b2 (mRNA vaccine) vaccination was unlikely to be associated with increased risks of incident hyperthyroidism or hypothyroidism. Thyroid dysfunction following COVID-19 vaccination was rare.

## Supplementary Information

**Additional file 1: Figure S1.** Histograms of number of thyroid dysfunction events by interval between vaccination date and event date by vaccines and doses. Pink area shows the 56 days after vaccination. **Figure S2.** Study flow diagram. **Table S1.** Baseline characteristics of people who experienced the individual outcomes by types of vaccine between 23 February 2021 and 30 September 2021. **Table S2.** Crude incidence rate and incidence of thyroid dysfunction within 56-day following the first or second dose of COVID-19 vaccination. **Table S3.** Risks of thyroid dysfunction in the 56-day risk period following the first or second dose of COVID-19 vaccination by age group among men (age <65 vs ≥65 years). **Table S4.** Risks of thyroid dysfunction in the 56-day risk period following the first or second dose of COVID-19 vaccination by age group among women (age <65 vs ≥65 years). **Table S5.** Risks of thyroid dysfunction in the 42-day risk period following the first or second dose of COVID-19 vaccination. **Table S6.** Risks of thyroid dysfunction in the 35-day risk period following the first or second dose of COVID-19 vaccination. **Table S7.** Risks of thyroid dysfunction in the 28-day risk period following the first or second dose of COVID-19 vaccination. **Table S8.** Risks of thyroid dysfunction in the 21-day risk period following the first or second dose of COVID-19 vaccination. **Table S9.** Risks of thyroid dysfunction in the 56-day risk period following the first or second dose of COVID-19 vaccination after restricting analysis to the period after vaccination.

## Data Availability

The data that support the findings of this study were extracted from the Hospital Authority database in Hong Kong. Restrictions apply to the availability of these data, which were used under license for this study. Data sharing is prohibited by the Hospital Authority. All the analysis codes that support the findings are available from the corresponding author upon reasonable requests.

## Abbreviations

ACE2: Angiotensin-converting enzyme 2
ASIA: Autoimmune/inflammatory syndrome induced by adjuvants
ATD: Anti-thyroid drug
BNF: British National Formulary
BNT162b2: mRNA COVID-19 vaccine
CI: Confidence intervals
CoronaVac: Inactivated COVID-19 vaccine
COVID-19: Coronavirus disease 2019
DH: Department of Health
EMRs: Electronic medical records
GD: Graves’ disease
HA: Hospital Authority
ICD-9: The International Classification of Diseases-9 codes
IRR: Incidence rate ratio
LT4: Levothyroxine
NA: Not available
SARS-CoV-2: Severe acute respiratory syndrome coronavirus 2
SCCS: Self-controlled case series
SD: Standard deviation
TSH: Thyroid-stimulating hormone
