# Supplementary material for: Risk of thyroid dysfunction associated with mRNA and inactivated COVID-19 vaccines: a population-based study of 2.3 million vaccine recipients
Source: BMC Med. 2022 Oct 14;20:339. doi: 10.1186/s12916-022-02548-1 (PMC9560718; doi:10.1186/s12916-022-02548-1)
Supplement: Supplementary file 1 — Additional file 1: Figure S1. Histograms of number of thyroid dysfunction events by interval between vaccination date and event date by vaccines and doses. Pink area shows the 56 days after vaccination. Figure S2. Study flow diagram. Table S1. Baseline characteristics of people who experienced the individual outcomes by types of vaccine between 23 February 2021 and 30 September 2021. Table S2. Crude incidence rate and incidence of thyroid dysfunction within 56-day following the first or second dose of COVID-19 vaccination. Table S3. Risks of thyroid dysfunction in the 56-day risk period following the first or second dose of COVID-19 vaccination by age group among men (age <65 vs ≥65 years). Table S4. Risks of thyroid dysfunction in the 56-day risk period following the first or second dose of COVID-19 vaccination by age group among women (age <65 vs ≥65 years). Table S5. Risks of thyroid dysfunction in the 42-day risk period following the first or second dose of COVID-19 vaccination. Table S6. Risks of thyroid dysfunction in the 35-day risk period following the first or second dose of COVID-19 vaccination. Table S7. Risks of thyroid dysfunction in the 28-day risk period following the first or second dose of COVID-19 vaccination. Table S8. Risks of thyroid dysfunction in the 21-day risk period following the first or second dose of COVID-19 vaccination. Table S9. Risks of thyroid dysfunction in the 56-day risk period following the first or second dose of COVID-19 vaccination after restricting analysis to the period after vaccination. [file 12916_2022_2548_MOESM1_ESM.docx]

**Additional file 1 of**

**Risk of thyroid dysfunction associated with mRNA and inactivated COVID-19 vaccines: a population-based study of 2.3 million vaccine recipients**

**List of authors:** Carlos King Ho Wong,^1,2,3,*^ David Tak Wai Lui,^4,*^ Xi Xiong,^1,*^ Celine Sze Ling Chui,^3,5,6^ Francisco Tsz Tsun Lai,^1,3^ Xue Li,^1,3,4^ Eric Yuk Fai Wan,^1,2,3^ Ching‐Lung Cheung,^1,3^ Chi Ho Lee,^4^ Yu-Cho Woo,^4^ Ivan Chi Ho Au,^1^ Matthew Shing Hin Chung,^1^ Franco Wing Tak Cheng,^1^ Kathryn Choon Beng Tan,^4^ Ian Chi Kei Wong,^1,3,7, 8^

*These first authors contributed equally to this article.

**Table of Contents**

[Figure S1 Histograms of number of thyroid dysfunction events by interval between vaccination date and event date by vaccines and doses. Pink area shows the 56 days after vaccination. 2](#_Toc106789378)

[Figure S2 Study flow diagram 4](#_Toc106789379)

[Table S1 Baseline characteristics of people who experienced the individual outcomes by types of vaccine between 23rd February 2021 and 30th September 2021. 5](#_Toc106789380)

[Table S2 Crude incidence rate and incidence of thyroid dysfunction events within 56-day following the first or second dose of COVID-19 vaccination. 7](#_Toc106789381)

[Table S3 Risks of thyroid dysfunction in the 56-day risk period following the first or second dose of COVID-19 vaccination by age group among men (age <65 vs ≥65 years) 8](#_Toc106789382)

[Table S4 Risks of thyroid dysfunction in the 56-day risk period following the first or second dose of COVID-19 vaccination by age group among women (age <65 vs ≥65 years) 9](#_Toc106789383)

[Table S5 Risks of thyroid dysfunction in the 42-day risk period following the first or second dose of COVID-19 vaccination. 11](#_Toc106789384)

[Table S6 Risks of thyroid dysfunction in the 35-day risk period following the first or second dose of COVID-19 vaccination. 12](#_Toc106789385)

[Table S7 Risks of thyroid dysfunction in the 28-day risk period following the first or second dose of COVID-19 vaccination. 13](#_Toc106789386)

[Table S8 Risks of thyroid dysfunction in the 21-day risk period following the first or second dose of COVID-19 vaccination. 14](#_Toc106789387)

[Table S9 Risks of thyroid dysfunction in the 56-day risk period following the first or second dose of COVID-19 vaccination after restricting analysis to the period after vaccination. 15](#_Toc106789388)

# Figure S1. Histograms of number of thyroid dysfunction events by interval between vaccination date and event date by vaccines and doses. Pink area shows the 56 days after vaccination.


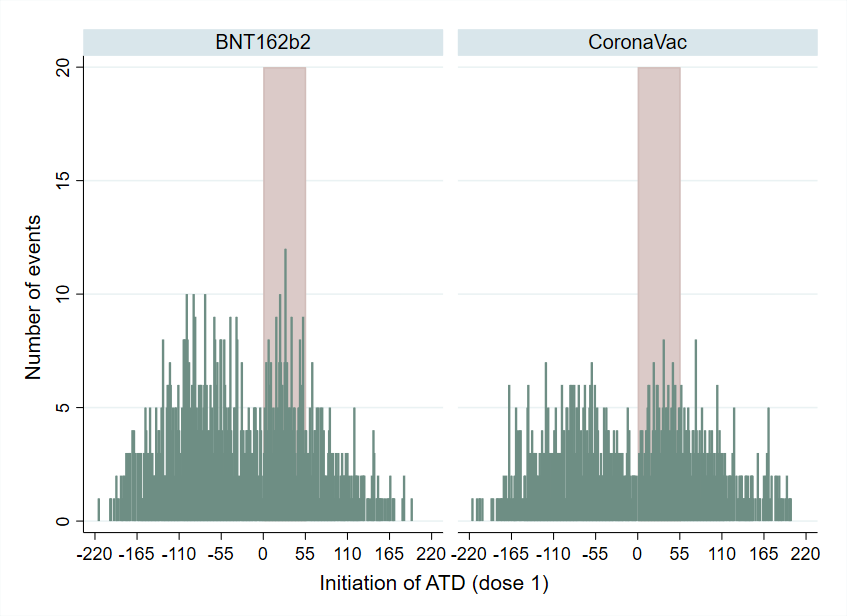

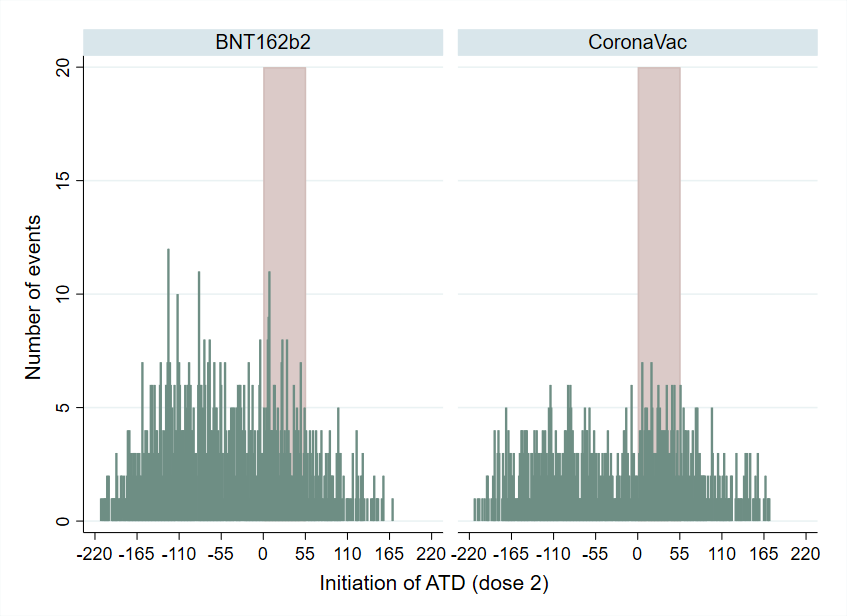


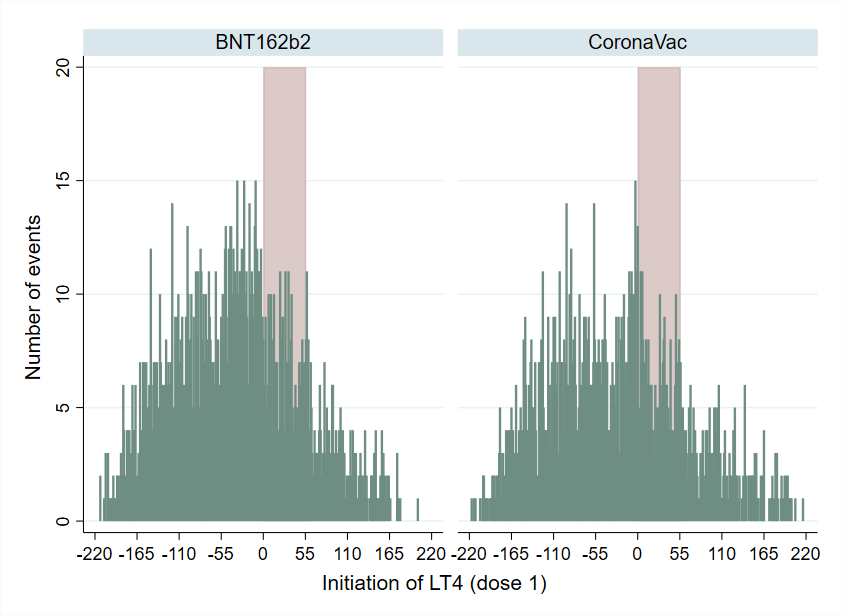

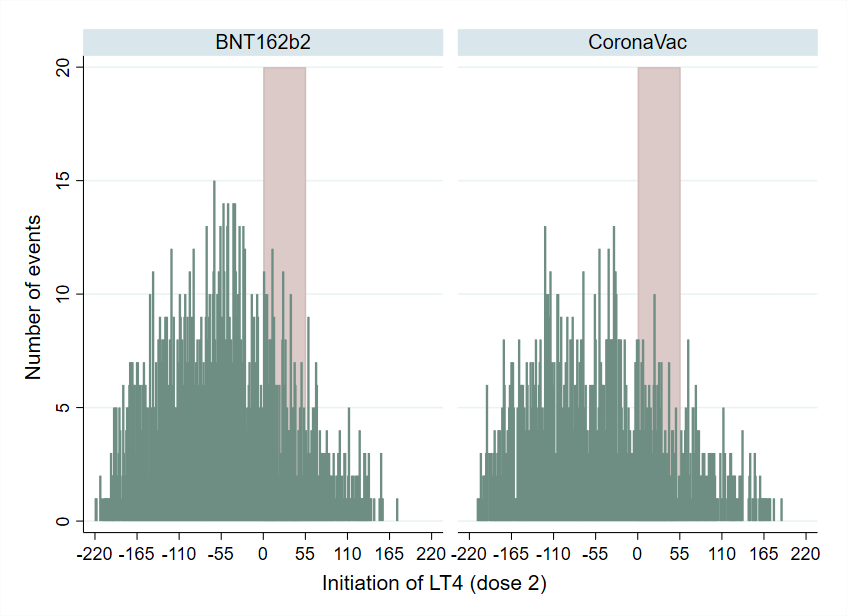


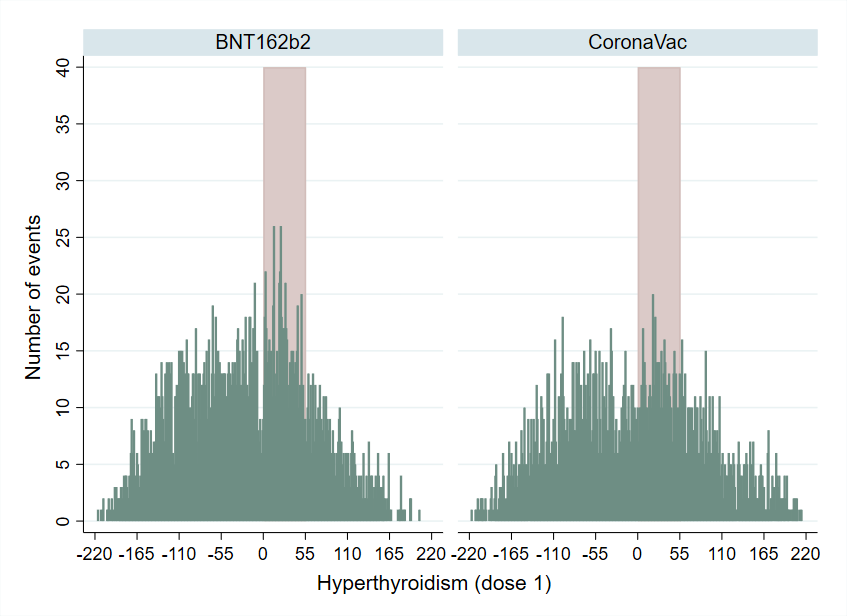

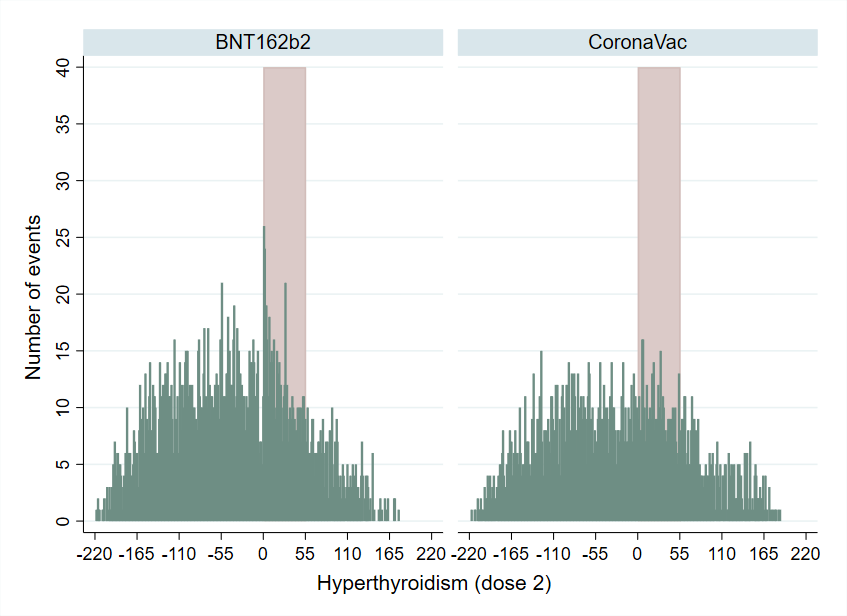


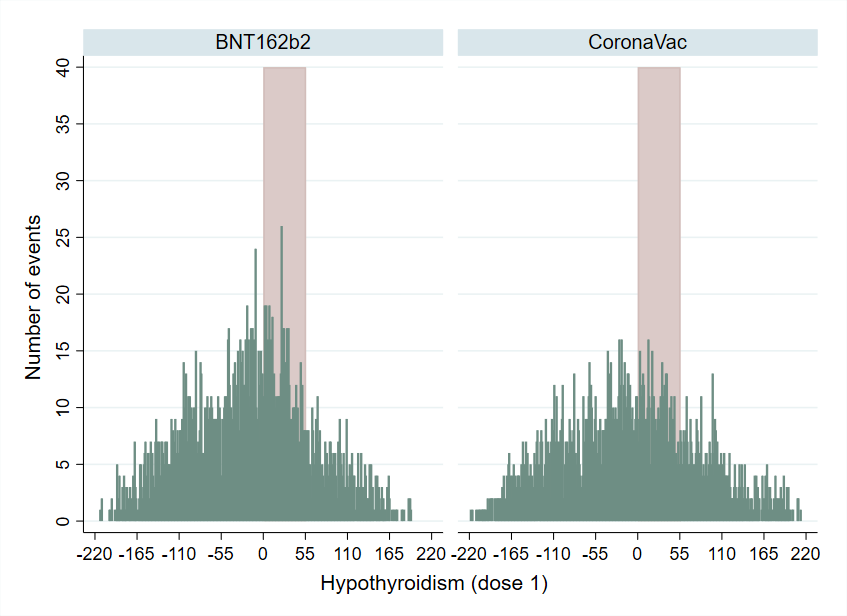

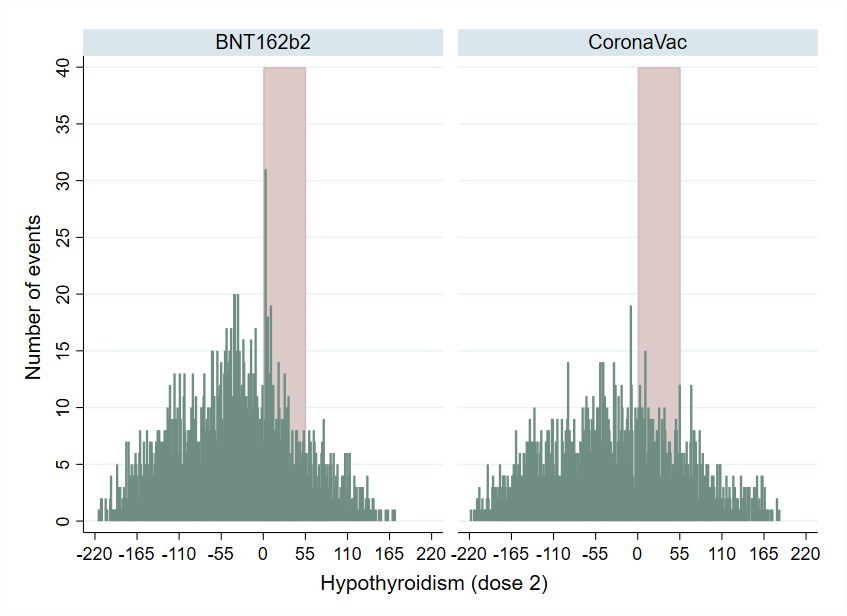


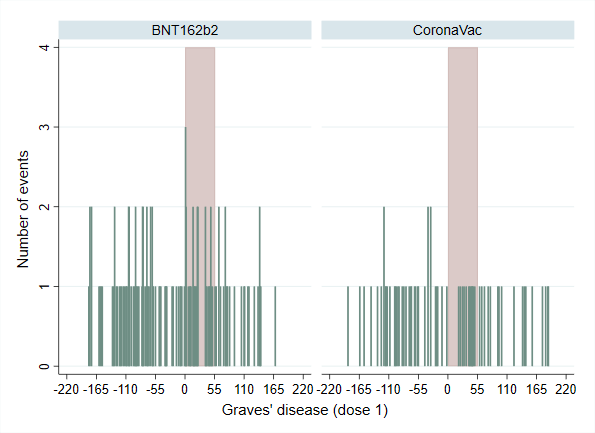

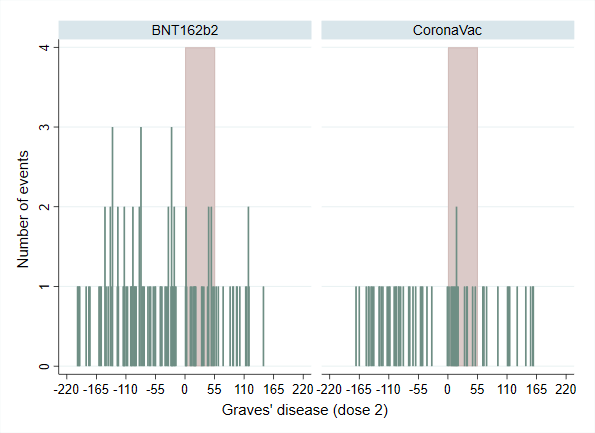


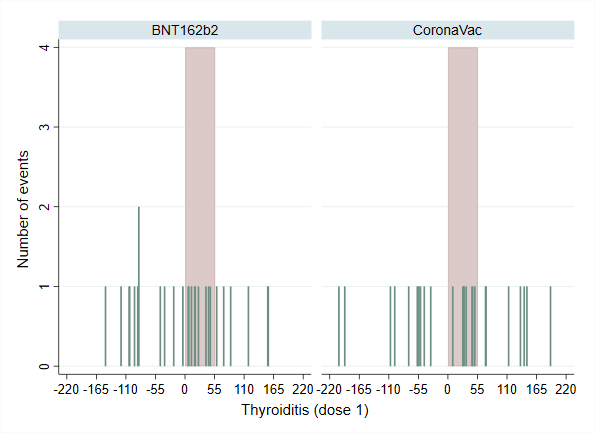

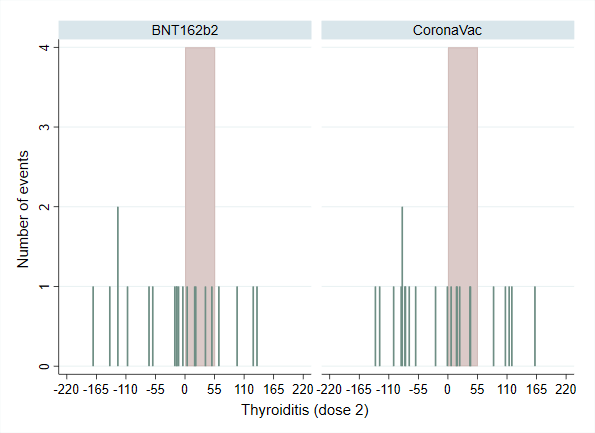


# Figure S2. Study flow diagram

* Unvaccinated patients with thyroid dysfunction during the observation period (e.g. no scheduled vaccination appointment, or cancellation of vaccination appointment if the thyroid dysfunction events occurred before the scheduled appointment) was included to inform the timing of events by adjusting for the monthly seasonal effects (35). These unvaccinated patients did not act as controls.

Note: ATD = anti-thyroid drug; LT4 = levothyroxine; TSH = thyroid-stimulating hormone

# Table S1. Baseline characteristics of people who experienced the individual outcomes by types of vaccine between 23rd February 2021 and 30th September 2021.

| Baseline characteristics | Initiation of ATD  (N = 4,594) | | Initiation of LT4  (N = 7,637) | | Hyperthyroidism   (N = 15,465) | | Hyperthyroidism   (N = 11,927) | | Graves' disease  (N = 434) | | Thyroiditis  (N = 97) | |
| --- | --- | --- | --- | --- | --- | --- | --- | --- | --- | --- | --- | --- |
|  | CoronaVac (N = 949) | BNT162b2 (N = 1,187) | CoronaVac (N = 1,630) | BNT162b2 (N = 1,938) | CoronaVac (N = 2,720) | BNT162b2 (N = 3,183) | CoronaVac (N = 2,216) | BNT162b2 (N = 2,540) | CoronaVac (N = 67) | BNT162b2 (N = 128) | CoronaVac (N = 26) | BNT162b2 (N = 27) |
| Age, years (Mean±SD) | 51.4±12.6 | 44.4±14.0 | 57.4±12.1 | 53.7±13.7 | 56.2±13.7 | 48.6±15.4 | 58.2±13.7 | 50.7±16.2 | 48.3±12.4 | 43.5±13.3 | 50.7±11.7 | 40.8±12.9 |
| 18-44 | 27.5% | 51.8% | 14.5% | 25.2% | 19.3% | 41.1% | 15.2% | 34.1% | 38.8% | 49.2% | 23.1% | 66.7% |
| 45-64 | 58.3% | 40.1% | 57.6% | 52.3% | 53.9% | 43.0% | 52.0% | 43.9% | 56.7% | 44.5% | 65.4% | 29.6% |
| ≥65 | 14.2% | 8.1% | 27.9% | 22.5% | 26.8% | 16.0% | 32.9% | 22.0% | 4.5% | 6.3% | 11.5% | 3.7% |
| Sex |  |  |  |  |  |  |  |  |  |  |  |  |
| Male | 27.0% | 24.2% | 24.5% | 22.5% | 33.8% | 28.7% | 30.1% | 27.8% | 26.9% | 21.9% | 19.2% | 14.8% |
| Female | 73.0% | 75.8% | 75.5% | 77.5% | 66.2% | 71.3% | 69.9% | 72.2% | 73.1% | 78.1% | 80.8% | 85.2% |
| Received the second dose | 85.0% | 88.5% | 88.1% | 92.3% | 85.6% | 89.3% | 85.6% | 90.7% | 88.1% | 85.2% | 88.5% | 77.8% |
| Pre-existing comorbidities |  |  |  |  |  |  |  |  |  |  |  |  |
| Charlson comorbidity index (Mean±SD) | 1.8±1.4 | 1.2±1.2 | 2.4±1.5 | 2.0±1.5 | 2.3±1.6 | 1.6±1.6 | 2.5±1.6 | 1.9±1.7 | 1.4±1.2 | 1.0±1.1 | 1.9±1.4 | 1.0±1.1 |
| 0 | 19.1% | 39.8% | 7.8% | 17.2% | 12.8% | 31.5% | 9.3% | 26.2% | 31.3% | 38.3% | 15.4% | 40.7% |
| 1-2 | 53.7% | 46.0% | 47.9% | 45.8% | 44.6% | 43.0% | 43.2% | 39.1% | 49.3% | 51.6% | 50.0% | 44.4% |
| ≥3 | 27.2% | 14.2% | 44.3% | 37.1% | 42.5% | 25.5% | 47.5% | 34.7% | 19.4% | 10.2% | 34.6% | 14.8% |
| Comorbidities |  |  |  |  |  |  |  |  |  |  |  |  |
| Myocardial infarction | 0.1% | 0.0% | 0.2% | 0.0% | 0.4% | 0.2% | 0.5% | 0.2% | 0.0% | 0.0% | 0.0% | 0.0% |
| Peripheral vascular disease | 0.0% | 0.0% | 0.2% | 0.0% | 0.2% | 0.1% | 0.0% | 0.0% | 0.0% | 0.0% | 0.0% | 0.0% |
| Cerebrovascular disease | 0.6% | 0.3% | 1.2% | 1.1% | 1.9% | 1.1% | 2.4% | 1.6% | 0.0% | 0.0% | 0.0% | 0.0% |
| Chronic obstructive pulmonary disease | 0.3% | 1.3% | 1.0% | 1.2% | 1.9% | 1.5% | 1.3% | 1.5% | 0.0% | 1.6% | 0.0% | 0.0% |
| Dementia | 0.1% | 0.0% | 0.1% | 0.0% | 0.2% | 0.0% | 0.1% | 0.1% | 0.0% | 0.0% | 0.0% | 0.0% |
| Paralysis | 0.1% | 0.0% | 0.0% | 0.0% | 0.0% | 0.0% | 0.0% | 0.0% | 0.0% | 0.0% | 0.0% | 0.0% |
| Diabetes without chronic complication | 5.5% | 2.7% | 5.6% | 3.6% | 8.5% | 5.0% | 8.3% | 5.9% | 0.0% | 0.8% | 23.1% | 0.0% |
| Diabetes with chronic complication | 0.2% | 0.1% | 0.2% | 0.1% | 0.6% | 0.2% | 0.5% | 0.6% | 1.5% | 0.0% | 0.0% | 0.0% |
| Chronic renal failure | 0.3% | 0.2% | 0.6% | 0.3% | 0.6% | 0.5% | 1.1% | 0.9% | 0.0% | 0.0% | 0.0% | 0.0% |
| Mild liver disease | 0.0% | 0.0% | 0.1% | 0.1% | 0.1% | 0.1% | 0.1% | 0.0% | 0.0% | 0.0% | 0.0% | 0.0% |
| Moderate-severe liver disease | 0.0% | 0.0% | 0.1% | 0.1% | 0.1% | 0.1% | 0.1% | 0.0% | 0.0% | 0.0% | 0.0% | 0.0% |
| Ulcers | 0.3% | 0.2% | 0.7% | 0.3% | 0.8% | 0.3% | 0.7% | 0.5% | 1.5% | 0.0% | 0.0% | 0.0% |
| Malignancy | 1.1% | 0.6% | 3.4% | 3.9% | 2.1% | 2.3% | 3.4% | 3.9% | 0.0% | 0.0% | 0.0% | 7.4% |
| Metastatic solid tumour | 0.2% | 0.0% | 0.6% | 0.3% | 0.3% | 0.2% | 0.4% | 0.5% | 0.0% | 0.0% | 0.0% | 0.0% |
| Drugs |  |  |  |  |  |  |  |  |  |  |  |  |
| Renin-angiotensin-system agents | 5.6% | 3.5% | 7.0% | 5.0% | 12.5% | 7.9% | 10.9% | 9.0% | 3.0% | 3.1% | 0.0% | 3.7% |
| Beta blockers | 3.5% | 2.1% | 5.1% | 3.9% | 7.7% | 4.9% | 8.8% | 7.4% | 4.5% | 1.6% | 3.8% | 0.0% |
| Calcium channel blockers | 10.4% | 6.9% | 12.8% | 8.6% | 18.3% | 11.2% | 17.0% | 13.7% | 6.0% | 1.6% | 7.7% | 3.7% |
| Diuretics | 1.7% | 0.8% | 1.7% | 1.2% | 2.3% | 1.9% | 2.9% | 3.0% | 1.5% | 0.0% | 0.0% | 0.0% |
| Nitrates | 0.5% | 0.4% | 0.8% | 0.7% | 1.7% | 1.1% | 1.7% | 1.4% | 0.0% | 0.8% | 0.0% | 0.0% |
| Lipid lowering agents | 8.7% | 4.3% | 11.6% | 8.2% | 16.6% | 10.6% | 17.6% | 15.4% | 4.5% | 3.1% | 19.2% | 0.0% |
| Antidiabetic drugs | 4.5% | 3.0% | 5.1% | 3.1% | 8.8% | 5.9% | 8.6% | 6.8% | 3.0% | 2.3% | 23.1% | 0.0% |
| Antiplatelets | 2.2% | 1.2% | 3.2% | 2.8% | 6.5% | 4.0% | 6.8% | 4.8% | 0.0% | 1.6% | 3.8% | 0.0% |
| Antidepressants | 2.0% | 1.9% | 4.1% | 3.9% | 4.4% | 4.3% | 6.8% | 6.4% | 1.5% | 0.8% | 0.0% | 0.0% |
| NSAIDs | 6.0% | 5.4% | 5.3% | 5.8% | 7.0% | 7.4% | 6.5% | 7.0% | 6.0% | 6.3% | 7.7% | 7.4% |
| Drugs for gout | 0.6% | 0.3% | 1.3% | 1.1% | 1.3% | 0.9% | 2.9% | 1.7% | 0.0% | 0.8% | 0.0% | 0.0% |

Note: ATD = anti-thyroid drug; LT4 = levothyroxine; NSAIDs = non-steroidal anti-inflammatory drugs; SD = standard deviation.

# Table S2. Crude incidence rate and incidence of thyroid dysfunction events within 56-day following the first or second dose of COVID-19 vaccination.

| Events | CoronaVac | | | | | BNT16212 | | | | |
| --- | --- | --- | --- | --- | --- | --- | --- | --- | --- | --- |
|  | Cases with event | Incidence rate  (Events / 100,000 person-years) | | Incidence (Events / 100,000 doses) | | Cases with event | Incidence rate  (Events / 100,000 person-years) | | Incidence (Events / 100,000 doses) | |
|  |  | Estimate | 95% CI | Estimate | 95% CI |  | Estimate | 95% CI | Estimate | 95% CI |
| **Initiation of ATD** | 653 | 354.1 | (327.5, 382.4) | 35.1 | (32.5, 37.9) | 815 | 357.0 | (332.9, 382.3) | 31.7 | (29.6, 34.0) |
| 0 to 55 days: first dose | 243 | 336.4 | (295.4, 381.5) | 25.1 | (22.1, 28.5) | 299 | 382.5 | (340.4, 428.4) | 22.6 | (20.1, 25.3) |
| 0 to 55 days: second dose | 410 | 365.6 | (331.0, 402.7) | 46.0 | (41.6, 50.7) | 516 | 343.6 | (314.6, 374.6) | 41.4 | (37.9, 45.1) |
| **Initiation of LT4** | 762 | 413.3 | (384.4, 443.7) | 41.0 | (38.2, 44.0) | 882 | 386.3 | (361.2, 412.7) | 34.3 | (32.1, 36.7) |
| 0 to 55 days: first dose | 321 | 444.4 | (397.1, 495.8) | 33.2 | (29.7, 37.1) | 281 | 359.5 | (318.7, 404.1) | 21.3 | (18.8, 23.9) |
| 0 to 55 days: second dose | 441 | 393.2 | (357.4, 431.7) | 49.5 | (45.0, 54.3) | 601 | 400.3 | (368.9, 433.6) | 48.2 | (44.4, 52.2) |
| **Hyperthyroidism (TSH <0.35 mIU/L)** | 1,113 | 603.7 | (568.7, 640.2) | 59.9 | (56.4, 63.5) | 1,289 | 564.6 | (534.2, 596.3) | 50.2 | (47.5, 53.0) |
| 0 to 55 days: first dose | 458 | 634.1 | (577.4, 694.9) | 47.4 | (43.1, 51.9) | 468 | 598.8 | (545.8, 655.6) | 35.4 | (32.3, 38.8) |
| 0 to 55 days: second dose | 655 | 584.1 | (540.2, 630.6) | 73.5 | (67.9, 79.3) | 821 | 546.8 | (510.1, 585.6) | 65.9 | (61.5, 70.5) |
| **Hypothyroidism (TSH >4.8 mIU/L)** | 820 | 444.7 | (414.8, 476.2) | 44.1 | (41.2, 47.3) | 935 | 409.5 | (383.7, 436.6) | 36.4 | (34.1, 38.8) |
| 0 to 55 days: first dose | 356 | 492.9 | (443.0, 546.8) | 36.8 | (33.1, 40.9) | 350 | 447.8 | (402.1, 497.3) | 26.5 | (23.8, 29.4) |
| 0 to 55 days: second dose | 464 | 413.7 | (376.9, 453.1) | 52.0 | (47.4, 57.0) | 585 | 389.6 | (358.7, 422.5) | 46.9 | (43.2, 50.9) |
| **Graves' disease** | 21 | 11.4 | (7.0, 17.4) | 1.1 | (0.7, 1.7) | 43 | 18.8 | (13.6, 25.4) | 1.7 | (1.2, 2.3) |
| 0 to 55 days: first dose | 4 | 5.5 | (1.5, 14.2) | 0.4 | (0.1, 1.1) | 21 | 26.9 | (16.6, 41.1) | 1.6 | (1.0, 2.4) |
| 0 to 55 days: second dose | 17 | 15.2 | (8.8, 24.3) | 1.9 | (1.1, 3.1) | 22 | 14.6 | (9.2, 22.2) | 1.8 | (1.1, 2.7) |
| **Thyroiditis** | 10 | 5.4 | (2.6, 10.0) | 0.5 | (0.3, 1.0) | 11 | 4.8 | (2.4, 8.6) | 0.4 | (0.2, 0.8) |
| 0 to 55 days: first dose | 4 | 5.5 | (1.5, 14.2) | 0.4 | (0.1, 1.1) | 6 | 7.7 | (2.8, 16.7) | 0.5 | (0.2, 1.0) |
| 0 to 55 days: second dose | 6 | 5.3 | (2.0, 11.6) | 0.7 | (0.2, 1.5) | 5 | 3.3 | (1.1, 7.8) | 0.4 | (0.1, 0.9) |

Note: ATD = anti-thyroid drug; LT4 = levothyroxine; CI = confidence interval; TSH = thyroid-stimulating hormone.

# Table S3. Risks of thyroid dysfunction in the 56-day risk period following the first or second dose of COVID-19 vaccination by age group among men (age <65 vs ≥65 years)

| **Outcomes** | **CoronaVac** | | | | **BNT162b2** | | | |
| --- | --- | --- | --- | --- | --- | --- | --- | --- |
|  | **No. of event** | **Person years** | **IRR** | **95% CI** | **No. of event** | **Person years** | **IRR** | **95% CI** |
| **Age < 65 years** |  |  |  |  |  |  |  |  |
| **Initiation of ATD** |  |  |  |  |  |  |  |  |
| Baseline | 532 | 316.8 | 1.00 | - | 558 | 339.3 | 1.00 | - |
| 0 to 55 days: first dose | 26 | 16.4 | 0.856 | (0.518, 1.415) | 27 | 16.2 | 1.045 | (0.618, 1.766) |
| 0 to 55 days: second dose | 31 | 21.6 | 0.788 | (0.467, 1.331) | 49 | 26.4 | 1.109 | (0.674, 1.824) |
| **Initiation of LT4** |  |  |  |  |  |  |  |  |
| Baseline | 675 | 401.6 | 1.00 | - | 735 | 425.7 | 1.00 | - |
| 0 to 55 days: first dose | 36 | 20.2 | 1.214 | (0.744, 1.981) | 22 | 18.9 | 0.803 | (0.449, 1.438) |
| 0 to 55 days: second dose | 36 | 28.1 | 0.942 | (0.562, 1.581) | 42 | 36.6 | 0.752 | (0.465, 1.213) |
| **Hyperthyroidism (TSH <0.35 mIU/L)** | | | | |  |  |  |  |
| Baseline | 1793 | 1091.6 | 1.00 | - | 1844 | 1135.3 | 1.00 | - |
| 0 to 55 days: first dose | 88 | 50.7 | 0.885 | (0.654, 1.198) | 84 | 45.4 | 1.005 | (0.742, 1.362) |
| 0 to 55 days: second dose | 126 | 66.6 | 1.005 | (0.758, 1.334) | 160 | 76.9 | 1.279 | (0.962, 1.702) |
| **Hypothyroidism (TSH >4.8 mIU/L)** | | | | |  |  |  |  |
| Baseline | 1136 | 690.0 | 1.00 | - | 1221 | 744.3 | 1.00 | - |
| 0 to 55 days: first dose | 52 | 28.4 | 1.014 | (0.680, 1.513) | 64 | 30.1 | 1.032 | (0.708, 1.506) |
| 0 to 55 days: second dose | 67 | 37.5 | 1.040 | (0.700, 1.544) | 92 | 55.0 | 0.857 | (0.604, 1.214) |
| **Graves' disease** |  |  |  |  |  |  |  |  |
| Baseline | 64 | 37.8 | 1.00 | - | 64 | 40.7 | 1.00 | - |
| 0 to 55 days: first dose | 1 | 1.4 | NA | NA | 6 | 1.5 | NA | NA |
| 0 to 55 days: second dose | 3 | 1.7 | NA | NA | 3 | 1.8 | NA | NA |
| **Thyroiditis** |  |  |  |  |  |  |  |  |
| Baseline | 8 | 4.5 | 1.00 | - | 8 | 4.8 | 1.00 | - |
| 0 to 55 days: first dose | 0 | 0.3 | NA | NA | 0 | 0.2 | NA | NA |
| 0 to 55 days: second dose | 1 | 0.6 | NA | NA | 1 | 0.5 | NA | NA |
|  |  |  |  |  |  |  |  |  |
| **Age ≥ 65 years** |  |  |  |  |  |  |  |  |
| **Initiation of ATD** |  |  |  |  |  |  |  |  |
| Baseline | 246 | 150.5 | 1.00 | - | 244 | 145.0 | 1.00 | - |
| 0 to 55 days: first dose | 10 | 4.3 | 1.467 | (0.551, 3.907) | 1 | 2.1 | NA | NA |
| 0 to 55 days: second dose | 9 | 4.8 | 1.144 | (0.349, 3.756) | 6 | 4.0 | NA | NA |
| **Initiation of LT4** |  |  |  |  |  |  |  |  |
| Baseline | 891 | 535.8 | 1.00 | - | 883 | 530.7 | 1.00 | - |
| 0 to 55 days: first dose | 17 | 10.5 | 0.763 | (0.364, 1.598) | 12 | 7.4 | 1.424 | (0.519, 3.912) |
| 0 to 55 days: second dose | 23 | 14.5 | 0.754 | (0.380, 1.497) | 20 | 13.0 | 1.546 | (0.680, 3.514) |
| **Hyperthyroidism (TSH <0.35 mIU/L)** | | | |  |  |  |  |  |
| Baseline | 2201 | 1326.5 | 1.00 | - | 2144 | 1293.8 | 1.00 | - |
| 0 to 55 days: first dose | 42 | 23.4 | 0.830 | (0.549, 1.253) | 24 | 12.4 | 0.771 | (0.421, 1.414) |
| 0 to 55 days: second dose | 48 | 30.1 | 0.777 | (0.501, 1.207) | 34 | 20.1 | 0.836 | (0.471, 1.483) |
| **Hypothyroidism (TSH >4.8 mIU/L)** | | | | |  |  |  |  |
| Baseline | 2184 | 1323.2 | 1.00 | - | 2152 | 1290.7 | 1.00 | - |
| 0 to 55 days: first dose | 46 | 23.5 | 1.001 | (0.632, 1.586) | 26 | 13.5 | 0.829 | (0.455, 1.512) |
| 0 to 55 days: second dose | 58 | 31.5 | 1.012 | (0.659, 1.556) | 26 | 23.3 | 0.496 | (0.284, 0.865) |
| **Graves' disease** |  |  |  |  |  |  |  |  |
| Baseline | 13 | 7.6 | 1.00 | - | 18 | 9.6 | 1.00 | - |
| 0 to 55 days: first dose | 0 | 0.1 | NA | NA | 0 | 0.4 | NA | NA |
| 0 to 55 days: second dose | 0 | 0.2 | NA | NA | 0 | 0.8 | NA | NA |
| **Thyroiditis** |  |  |  |  |  |  |  |  |
| Baseline | 6 | 3.4 | 1.00 | - | 5 | 3.0 | 1.00 | - |
| 0 to 55 days: first dose | 0 | 0.1 | NA | NA | 0 | 0.0 | NA | NA |
| 0 to 55 days: second dose | 0 | 0.2 | NA | NA | 0 | 0.0 | NA | NA |

Note: ATD = anti-thyroid drug; LT4 = levothyroxine; IRR = incidence rate ratio; CI = confidence interval; TSH = thyroid-stimulating hormone; NA = not available if the number of events in one of the risk periods were ≤ 5.

# Table S4. Risks of thyroid dysfunction in the 56-day risk period following the first or second dose of COVID-19 vaccination by age group among women (age <65 vs ≥65 years)

| **Outcomes** | **CoronaVac** | | | | **BNT162b2** | | | |
| --- | --- | --- | --- | --- | --- | --- | --- | --- |
|  | **No. of event** | **Person years** | **IRR** | **95% CI** | **No. of event** | **Person years** | **IRR** | **95% CI** |
| **Age < 65 years** |  |  |  |  |  |  |  |  |
| **Initiation of ATD** |  |  |  |  |  |  |  |  |
| Baseline | 1795 | 1087.5 | 1.00 | - | 1971 | 1201.3 | 1.00 | - |
| 0 to 55 days: first dose | 64 | 47.2 | 0.636 | (0.457, 0.885) | 97 | 54.5 | 0.877 | (0.652, 1.179) |
| 0 to 55 days: second dose | 131 | 63.9 | 0.968 | (0.719, 1.303) | 154 | 82.6 | 1.000 | (0.761, 1.314) |
| **Initiation of LT4** |  |  |  |  |  |  |  |  |
| Baseline | 2258 | 1311.0 | 1.00 | - | 2466 | 1439.1 | 1.00 | - |
| 0 to 55 days: first dose | 80 | 71.4 | 0.677 | (0.488, 0.941) | 97 | 73.5 | 0.933 | (0.691, 1.260) |
| 0 to 55 days: second dose | 125 | 101.1 | 0.777 | (0.571, 1.058) | 174 | 135.9 | 0.947 | (0.732, 1.225) |
| **Hyperthyroidism (TSH <0.35 mIU/L)** | | | | |  |  |  |  |
| Baseline | 4128 | 2490.1 | 1.00 | - | 4572 | 2778.0 | 1.00 | - |
| 0 to 55 days: first dose | 175 | 107.1 | 0.839 | (0.674, 1.045) | 217 | 126.3 | 0.865 | (0.709, 1.055) |
| 0 to 55 days: second dose | 258 | 150.0 | 0.879 | (0.712, 1.085) | 376 | 206.7 | 1.001 | (0.834, 1.200) |
| **Hypothyroidism (TSH >4.8 mIU/L)** | | | | |  |  |  |  |
| Baseline | 2653 | 1602.1 | 1.00 | - | 2908 | 1783.0 | 1.00 | - |
| 0 to 55 days: first dose | 151 | 88.4 | 0.970 | (0.754, 1.246) | 188 | 94.9 | 1.096 | (0.871, 1.378) |
| 0 to 55 days: second dose | 207 | 123.2 | 1.004 | (0.793, 1.270) | 287 | 159.8 | 1.069 | (0.862, 1.326) |
| **Graves' disease** |  |  |  |  |  |  |  |  |
| Baseline | 180 | 107.0 | 1.00 | - | 211 | 129.7 | 1.00 | - |
| 0 to 55 days: first dose | 1 | 3.5 | NA | NA | 15 | 7.0 | 1.322 | (0.572, 3.056) |
| 0 to 55 days: second dose | 11 | 5.2 | NA | NA | 17 | 9.7 | 1.082 | (0.482, 2.429) |
| **Thyroiditis** |  |  |  |  |  |  |  |  |
| Baseline | 34 | 21.5 | 1.00 | - | 35 | 23.2 | 1.00 | - |
| 0 to 55 days: first dose | 3 | 1.5 | NA | NA | 6 | 1.5 | NA | NA |
| 0 to 55 days: second dose | 5 | 2.2 | NA | NA | 4 | 2.4 | NA | NA |
|  |  |  |  |  |  |  |  |  |
| **Age ≥ 65 years** |  |  |  |  |  |  |  |  |
| **Initiation of ATD** |  |  |  |  |  |  |  |  |
| Baseline | 543 | 323.5 | 1.00 | - | 526 | 314.4 | 1.00 | - |
| 0 to 55 days: first dose | 9 | 6.6 | 0.684 | (0.284, 1.649) | 5 | 3.6 | NA | NA |
| 0 to 55 days: second dose | 11 | 9.0 | 0.564 | (0.247, 1.283) | 7 | 6.0 | NA | NA |
| **Initiation of LT4** |  |  |  |  |  |  |  |  |
| Baseline | 1491 | 883.2 | 1.00 | - | 1491 | 884.8 | 1.00 | - |
| 0 to 55 days: first dose | 34 | 23.6 | 0.772 | (0.457, 1.304) | 13 | 18.7 | 0.726 | (0.356, 1.478) |
| 0 to 55 days: second dose | 33 | 31.7 | 0.625 | (0.359, 1.088) | 52 | 33.7 | 1.581 | (0.938, 2.664) |
| **Hyperthyroidism (TSH <0.35 mIU/L)** | | | | |  |  |  |  |
| Baseline | 3301 | 1988.7 | 1.00 | - | 3221 | 1934.3 | 1.00 | - |
| 0 to 55 days: first dose | 49 | 33.3 | 0.669 | (0.432, 1.036) | 20 | 17.5 | 0.575 | (0.287, 1.153) |
| 0 to 55 days: second dose | 73 | 39.8 | 0.941 | (0.625, 1.419) | 49 | 29.9 | 0.806 | (0.469, 1.386) |
| **Hypothyroidism (TSH >4.8 mIU/L)** | | | | |  |  |  |  |
| Baseline | 2734 | 1634.9 | 1.00 | - | 2660 | 1601.5 | 1.00 | - |
| 0 to 55 days: first dose | 51 | 32.1 | 0.899 | (0.575, 1.406) | 29 | 20.4 | 0.785 | (0.426, 1.446) |
| 0 to 55 days: second dose | 48 | 39.4 | 0.664 | (0.427, 1.034) | 58 | 32.7 | 0.958 | (0.575, 1.596) |
| **Graves' disease** |  |  |  |  |  |  |  |  |
| Baseline | 31 | 19.5 | 1.00 | - | 32 | 19.5 | 1.00 | - |
| 0 to 55 days: first dose | 1 | 0.2 | NA | NA | 0 | 0.1 | NA | NA |
| 0 to 55 days: second dose | 1 | 0.2 | NA | NA | 1 | 0.3 | NA | NA |
| **Thyroiditis** |  |  |  |  |  |  |  |  |
| Baseline | 12 | 7.6 | 1.00 | - | 12 | 7.2 | 1.00 | - |
| 0 to 55 days: first dose | 1 | 0.2 | NA | NA | 0 | 0.0 | NA | NA |
| 0 to 55 days: second dose | 0 | 0.0 | NA | NA | 0 | 0.0 | NA | NA |

Note: ATD = anti-thyroid drug; LT4 = levothyroxine; IRR = incidence rate ratio; CI = confidence interval; TSH = thyroid-stimulating hormone; NA = not available if the number of events in one of the risk periods were ≤ 5.

# Table S5. Risks of thyroid dysfunction in the 42-day risk period following the first or second dose of COVID-19 vaccination.

| **Outcomes** | **CoronaVac** | | | | **BNT162b2** | | | |
| --- | --- | --- | --- | --- | --- | --- | --- | --- |
|  | **No. of event** | **Person years** | **IRR** | **95% CI** | **No. of event** | **Person years** | **IRR** | **95% CI** |
| **Initiation of ATD** |  |  |  |  |  |  |  |  |
| Baseline | 3158 | 1899.8 | 1.00 | - | 3348 | 2022.6 | 1.00 | - |
| 0 to 41 days: first dose | 107 | 72.8 | 0.776 | (0.609, 0.989) | 124 | 74.9 | 0.851 | (0.666, 1.086) |
| 0 to 41 days: second dose | 142 | 79.6 | 0.912 | (0.716, 1.161) | 173 | 98.0 | 0.962 | (0.764, 1.213) |
|  |  |  |  |  |  |  |  |  |
| **Initiation of LT4** |  |  |  |  |  |  |  |  |
| Baseline | 5351 | 3167.7 | 1.00 | - | 5622 | 3323.3 | 1.00 | - |
| 0 to 41 days: first dose | 161 | 124.1 | 0.872 | (0.702, 1.084) | 142 | 117.7 | 0.980 | (0.782, 1.229) |
| 0 to 41 days: second dose | 187 | 140.9 | 0.947 | (0.767, 1.170) | 243 | 177.2 | 1.155 | (0.953, 1.399) |
|  |  |  |  |  |  |  |  |  |
| **Hyperthyroidism (TSH <0.35 mIU/L)** | | | | |  |  |  |  |
| Baseline | 11548 | 6958.8 | 1.00 | - | 11901 | 7206.9 | 1.00 | - |
| 0 to 41 days: first dose | 342 | 209.1 | 0.837 | (0.722, 0.970) | 337 | 198.3 | 0.891 | (0.767, 1.036) |
| 0 to 41 days: second dose | 392 | 229.9 | 0.871 | (0.751, 1.011) | 507 | 271.5 | 1.065 | (0.926, 1.225) |
|  |  |  |  |  |  |  |  |  |
| **Hypothyroidism (TSH >4.8 mIU/L)** | | | | |  |  |  |  |
| Baseline | 8796 | 5299.3 | 1.00 | - | 9028 | 5472.2 | 1.00 | - |
| 0 to 41 days: first dose | 295 | 169.2 | 0.966 | (0.816, 1.143) | 301 | 156.6 | 1.042 | (0.879, 1.234) |
| 0 to 41 days: second dose | 296 | 185.6 | 0.912 | (0.773, 1.076) | 382 | 220.4 | 0.983 | (0.837, 1.154) |
|  |  |  |  |  |  |  |  |  |
| **Graves' disease** |  |  |  |  |  |  |  |  |
| Baseline | 290 | 173.4 | 1.00 | - | 336 | 202.0 | 1.00 | - |
| 0 to 41 days: first dose | 3 | 5.2 | NA | NA | 17 | 8.7 | 0.608 | (0.273, 1.352) |
| 0 to 41 days: second dose | 13 | 5.8 | NA | NA | 14 | 10.3 | 0.495 | (0.234, 1.044) |
|  |  |  |  |  |  |  |  |  |
| **Thyroiditis** |  |  |  |  |  |  |  |  |
| Baseline | 61 | 37.7 | 1.00 | - | 62 | 38.9 | 1.00 | - |
| 0 to 41 days: first dose | 4 | 2.0 | NA | NA | 5 | 1.7 | NA | NA |
| 0 to 41 days: second dose | 5 | 2.4 | NA | NA | 4 | 2.2 | NA | NA |

Note: ATD = anti-thyroid drug; LT4 = levothyroxine; IRR = incidence rate ratio; CI = confidence interval; TSH = thyroid-stimulating hormone; NA = not available if the number of events in one of the risk periods were ≤ 5.

# Table S6. Risks of thyroid dysfunction in the 35-day risk period following the first or second dose of COVID-19 vaccination.

| **Outcomes** | **CoronaVac** | | | | **BNT162b2** | | | |
| --- | --- | --- | --- | --- | --- | --- | --- | --- |
|  | **No. of event** | **Person years** | **IRR** | **95% CI** | **No. of event** | **Person years** | **IRR** | **95% CI** |
| **Initiation of ATD** |  |  |  |  |  |  |  |  |
| Baseline | 3187 | 1912.0 | 1.00 | - | 3368 | 2036.6 | 1.00 | - |
| 0 to 34 days: first dose | 103 | 71.6 | 0.766 | (0.600, 0.978) | 124 | 73.7 | 0.946 | (0.750, 1.193) |
| 0 to 34 days: second dose | 117 | 68.5 | 0.875 | (0.680, 1.125) | 153 | 85.2 | 1.040 | (0.831, 1.301) |
|  |  |  |  |  |  |  |  |  |
| **Initiation of LT4** |  |  |  |  |  |  |  |  |
| Baseline | 5374 | 3188.1 | 1.00 | - | 5662 | 3347.9 | 1.00 | - |
| 0 to 34 days: first dose | 158 | 123.1 | 0.900 | (0.728, 1.112) | 141 | 117.1 | 0.928 | (0.743, 1.158) |
| 0 to 34 days: second dose | 167 | 121.5 | 1.023 | (0.831, 1.260) | 204 | 153.2 | 1.060 | (0.873, 1.287) |
|  |  |  |  |  |  |  |  |  |
| **Hyperthyroidism (TSH <0.35 mIU/L)** | | | | |  |  |  |  |
| Baseline | 11603 | 6993.9 | 1.00 | - | 11973 | 7245.6 | 1.00 | - |
| 0 to 34 days: first dose | 338 | 205.8 | 0.891 | (0.771, 1.028) | 331 | 195.8 | 0.904 | (0.782, 1.045) |
| 0 to 34 days: second dose | 341 | 198.1 | 0.915 | (0.787, 1.064) | 441 | 235.3 | 1.071 | (0.934, 1.230) |
|  |  |  |  |  |  |  |  |  |
| **Hypothyroidism (TSH >4.8 mIU/L)** | | | | |  |  |  |  |
| Baseline | 8849 | 5327.1 | 1.00 | - | 9065 | 5502.9 | 1.00 | - |
| 0 to 34 days: first dose | 287 | 167.1 | 0.949 | (0.804, 1.120) | 301 | 155.0 | 1.141 | (0.971, 1.341) |
| 0 to 34 days: second dose | 251 | 159.9 | 0.898 | (0.758, 1.064) | 345 | 191.3 | 1.091 | (0.933, 1.276) |
|  |  |  |  |  |  |  |  |  |
| **Graves' disease** |  |  |  |  |  |  |  |  |
| Baseline | 292 | 174.2 | 1.00 | - | 337 | 203.6 | 1.00 | - |
| 0 to 34 days: first dose | 3 | 5.1 | NA | NA | 17 | 8.5 | 0.803 | (0.370, 1.743) |
| 0 to 34 days: second dose | 11 | 5.0 | NA | NA | 13 | 8.9 | 0.638 | (0.286, 1.421) |
|  |  |  |  |  |  |  |  |  |
| **Thyroiditis** |  |  |  |  |  |  |  |  |
| Baseline | 62 | 38.1 | 1.00 | - | 63 | 39.2 | 1.00 | - |
| 0 to 34 days: first dose | 4 | 2.0 | NA | NA | 5 | 1.7 | NA | NA |
| 0 to 34 days: second dose | 4 | 2.1 | NA | NA | 3 | 1.9 | NA | NA |

Note: ATD = anti-thyroid drug; LT4 = levothyroxine; IRR = incidence rate ratio; CI = confidence interval; TSH = thyroid-stimulating hormone; NA = not available if the number of events in one of the risk periods were ≤ 5.

# Table S7. Risks of thyroid dysfunction in the 28-day risk period following the first or second dose of COVID-19 vaccination.

| **Outcomes** | **CoronaVac** | | | | **BNT162b2** | | | |
| --- | --- | --- | --- | --- | --- | --- | --- | --- |
|  | **No. of event** | **Person years** | **IRR** | **95% CI** | **No. of event** | **Person years** | **IRR** | **95% CI** |
| **Initiation of ATD** |  |  |  |  |  |  |  |  |
| Baseline | 3223 | 1926.9 | 1.00 | - | 3399 | 2052.9 | 1.00 | - |
| 0 to 27 days: first dose | 91 | 68.8 | 0.704 | (0.545, 0.909) | 117 | 71.7 | 0.952 | (0.755, 1.201) |
| 0 to 27 days: second dose | 93 | 56.4 | 0.856 | (0.661, 1.109) | 129 | 70.8 | 1.083 | (0.860, 1.364) |
|  |  |  |  |  |  |  |  |  |
| **Initiation of LT4** |  |  |  |  |  |  |  |  |
| Baseline | 5412 | 3213.4 | 1.00 | - | 5695 | 3375.8 | 1.00 | - |
| 0 to 27 days: first dose | 150 | 119.2 | 0.865 | (0.699, 1.069) | 140 | 115.8 | 0.945 | (0.760, 1.173) |
| 0 to 27 days: second dose | 137 | 100.0 | 0.999 | (0.800, 1.249) | 172 | 126.5 | 1.087 | (0.890, 1.327) |
|  |  |  |  |  |  |  |  |  |
| **Hyperthyroidism (TSH <0.35 mIU/L)** | | | | |  |  |  |  |
| Baseline | 11700 | 7036.6 | 1.00 | - | 12050 | 7290.1 | 1.00 | - |
| 0 to 27 days: first dose | 315 | 197.9 | 0.854 | (0.739, 0.987) | 328 | 191.6 | 0.948 | (0.823, 1.093) |
| 0 to 27 days: second dose | 267 | 163.3 | 0.850 | (0.724, 0.998) | 367 | 194.9 | 1.073 | (0.931, 1.237) |
|  |  |  |  |  |  |  |  |  |
| **Hypothyroidism (TSH >4.8 mIU/L)** | | | | |  |  |  |  |
| Baseline | 8906 | 5361.2 | 1.00 | - | 9130 | 5538.3 | 1.00 | - |
| 0 to 27 days: first dose | 270 | 161.1 | 0.951 | (0.808, 1.118) | 294 | 152.3 | 1.137 | (0.971, 1.331) |
| 0 to 27 days: second dose | 211 | 131.8 | 0.952 | (0.801, 1.131) | 287 | 158.6 | 1.081 | (0.922, 1.267) |
|  |  |  |  |  |  |  |  |  |
| **Graves' disease** |  |  |  |  |  |  |  |  |
| Baseline | 293 | 175.3 | 1.00 | - | 339 | 205.6 | 1.00 | - |
| 0 to 27 days: first dose | 3 | 4.9 | NA | NA | 17 | 8.1 | 1.026 | (0.479, 2.198) |
| 0 to 27 days: second dose | 10 | 4.1 | NA | NA | 11 | 7.4 | 0.728 | (0.309, 1.716) |
|  |  |  |  |  |  |  |  |  |
| **Thyroiditis** |  |  |  |  |  |  |  |  |
| Baseline | 65 | 38.5 | 1.00 | - | 63 | 39.6 | 1.00 | - |
| 0 to 27 days: first dose | 1 | 1.9 | NA | NA | 5 | 1.6 | NA | NA |
| 0 to 27 days: second dose | 4 | 1.7 | NA | NA | 3 | 1.5 | NA | NA |

Note: ATD = anti-thyroid drug; LT4 = levothyroxine; IRR = incidence rate ratio; CI = confidence interval; TSH = thyroid-stimulating hormone; NA = not available if the number of events in one of the risk periods were ≤ 5.

# Table S8. Risks of thyroid dysfunction in the 21-day risk period following the first or second dose of COVID-19 vaccination.

| **Outcomes** | **CoronaVac** | | | | **BNT162b2** | | | |
| --- | --- | --- | --- | --- | --- | --- | --- | --- |
|  | **No. of event** | **Person years** | **IRR** | **95% CI** | **No. of event** | **Person years** | **IRR** | **95% CI** |
| **Initiation of ATD** |  |  |  |  |  |  |  |  |
| Baseline | 3276 | 1956.5 | 1.00 | - | 3448 | 2074.6 | 1.00 | - |
| 0 to 20 days: first dose | 60 | 52.3 | 0.626 | (0.470, 0.833) | 97 | 65.9 | 0.839 | (0.658, 1.070) |
| 0 to 20 days: second dose | 71 | 43.4 | 0.887 | (0.676, 1.164) | 100 | 55.0 | 1.066 | (0.831, 1.368) |
|  |  |  |  |  |  |  |  |  |
| **Initiation of LT4** |  |  |  |  |  |  |  |  |
| Baseline | 5473 | 3264.9 | 1.00 | - | 5745 | 3412.3 | 1.00 | - |
| 0 to 20 days: first dose | 126 | 90.6 | 0.977 | (0.789, 1.209) | 129 | 108.5 | 0.920 | (0.741, 1.143) |
| 0 to 20 days: second dose | 100 | 77.2 | 0.921 | (0.721, 1.177) | 133 | 97.3 | 1.059 | (0.856, 1.310) |
|  |  |  |  |  |  |  |  |  |
| **Hyperthyroidism (TSH <0.35 mIU/L)** | | | | |  |  |  |  |
| Baseline | 11855 | 7121.8 | 1.00 | - | 12155 | 7348.7 | 1.00 | - |
| 0 to 20 days: first dose | 226 | 150.3 | 0.822 | (0.705, 0.958) | 296 | 177.1 | 0.954 | (0.826, 1.102) |
| 0 to 20 days: second dose | 201 | 125.7 | 0.844 | (0.711, 1.002) | 294 | 150.9 | 1.121 | (0.965, 1.303) |
|  |  |  |  |  |  |  |  |  |
| **Hypothyroidism (TSH >4.8 mIU/L)** | | | | |  |  |  |  |
| Baseline | 9002 | 5429.8 | 1.00 | - | 9210 | 5584.3 | 1.00 | - |
| 0 to 20 days: first dose | 210 | 122.6 | 1.031 | (0.871, 1.219) | 268 | 141.8 | 1.136 | (0.973, 1.327) |
| 0 to 20 days: second dose | 175 | 101.6 | 1.089 | (0.911, 1.301) | 233 | 123.0 | 1.160 | (0.981, 1.371) |
|  |  |  |  |  |  |  |  |  |
| **Graves' disease** |  |  |  |  |  |  |  |  |
| Baseline | 295 | 177.4 | 1.00 | - | 340 | 208.1 | 1.00 | - |
| 0 to 20 days: first dose | 1 | 3.7 | NA | NA | 16 | 7.2 | 1.427 | (0.716, 2.844) |
| 0 to 20 days: second dose | 10 | 3.1 | NA | NA | 11 | 5.7 | 1.179 | (0.525, 2.645) |
|  |  |  |  |  |  |  |  |  |
| **Thyroiditis** |  |  |  |  |  |  |  |  |
| Baseline | 66 | 39.4 | 1.00 | - | 63 | 40.1 | 1.00 | - |
| 0 to 20 days: first dose | 1 | 1.5 | NA | NA | 5 | 1.5 | NA | NA |
| 0 to 20 days: second dose | 3 | 1.3 | NA | NA | 3 | 1.2 | NA | NA |

Note: ATD = anti-thyroid drug; LT4 = levothyroxine; IRR = incidence rate ratio; CI = confidence interval; TSH = thyroid-stimulating hormone; NA = not available if the number of events in one of the risk periods were ≤ 5.

# Table S9. Risks of thyroid dysfunction in the 56-day risk period following the first or second dose of COVID-19 vaccination after restricting analysis to the period after vaccination.

| **Outcomes** | **CoronaVac** | | | | **BNT162b2** | | | |
| --- | --- | --- | --- | --- | --- | --- | --- | --- |
|  | **No. of event** | **Person years** | **IRR** | **95% CI** | **No. of event** | **Person years** | **IRR** | **95% CI** |
| **Initiation of ATD** |  |  |  |  |  |  |  |  |
| Baseline | 2634 | 1561.9 | 1.00 | - | 2610 | 1547.5 | 1.00 | - |
| 0 to 55 days: first dose | 243 | 60.5 | 0.938 | (0.726, 1.211) | 299 | 57.6 | 1.125 | (0.872, 1.452) |
| 0 to 55 days: second dose | 308 | 88.2 | 1.024 | (0.808, 1.297) | 403 | 100.1 | 1.124 | (0.892, 1.416) |
|  |  |  |  |  |  |  |  |  |
| **Initiation of LT4** |  |  |  |  |  |  |  |  |
| Baseline | 4335 | 2572.3 | 1.00 | - | 4364 | 2580.1 | 1.00 | - |
| 0 to 55 days: first dose | 321 | 78.8 | 0.811 | (0.655, 1.005) | 281 | 70.8 | 0.824 | (0.665, 1.020) |
| 0 to 55 days: second dose | 385 | 125.2 | 0.783 | (0.642, 0.956) | 546 | 148.8 | 0.990 | (0.828, 1.185) |
|  |  |  |  |  |  |  |  |  |
| **Hyperthyroidism (TSH <0.35 mIU/L)** | | | | |  |  |  |  |
| Baseline | 10039 | 5983.2 | 1.00 | - | 9964 | 5934.4 | 1.00 | - |
| 0 to 55 days: first dose | 458 | 129.8 | 0.906 | (0.773, 1.062) | 468 | 108.8 | 0.980 | (0.829, 1.158) |
| 0 to 55 days: second dose | 578 | 191.4 | 0.911 | (0.782, 1.061) | 732 | 202.1 | 1.026 | (0.880, 1.195) |
|  |  |  |  |  |  |  |  |  |
| **Hypothyroidism (TSH >4.8 mIU/L)** | | | | |  |  |  |  |
| Baseline | 7526 | 4487.8 | 1.00 | - | 7498 | 4455.8 | 1.00 | - |
| 0 to 55 days: first dose | 356 | 96.0 | 0.948 | (0.784, 1.146) | 350 | 81.9 | 0.926 | (0.762, 1.124) |
| 0 to 55 days: second dose | 438 | 147.1 | 0.938 | (0.788, 1.116) | 560 | 157.5 | 0.940 | (0.793, 1.115) |
|  |  |  |  |  |  |  |  |  |
| **Graves' disease** |  |  |  |  |  |  |  |  |
| Baseline | 251 | 149.7 | 1.00 | - | 254 | 151.2 | 1.00 | - |
| 0 to 55 days: first dose | 4 | 2.8 | NA | NA | 21 | 4.7 | 0.999 | (0.410, 2.433) |
| 0 to 55 days: second dose | 17 | 4.2 | NA | NA | 22 | 6.9 | 0.799 | (0.381, 1.675) |
|  |  |  |  |  |  |  |  |  |
| **Thyroiditis** |  |  |  |  |  |  |  |  |
| Baseline | 49 | 29.1 | 1.00 | - | 48 | 28.2 | 1.00 | - |
| 0 to 55 days: first dose | 4 | 1.3 | NA | NA | 6 | 1.1 | NA | NA |
| 0 to 55 days: second dose | 6 | 1.9 | NA | NA | 5 | 2.0 | NA | NA |

Note: ATD = anti-thyroid drug; LT4 = levothyroxine; IRR = incidence rate ratio; CI = confidence interval; TSH = thyroid-stimulating hormone; NA = not available if the number of events in one of the risk periods were ≤ 5.
